# Expressed sequence tags from larval gut of the European corn borer (*Ostrinia nubilalis*): Exploring candidate genes potentially involved in *Bacillus thuringiensis *toxicity and resistance

**DOI:** 10.1186/1471-2164-10-286

**Published:** 2009-06-29

**Authors:** Chitvan Khajuria, Yu Cheng Zhu, Ming-Shun Chen, Lawrent L Buschman, Randall A Higgins, Jianxiu Yao, Andre LB Crespo, Blair D Siegfried, Subbaratnam Muthukrishnan, Kun Yan Zhu

**Affiliations:** 1 Department of Entomology, 123 Waters Hall, Kansas State University, Manhattan, KS 66506, USA; 2 Southern Insect Management Unit, USDA-ARS, 141 Experiment Station Road, Stoneville, MS 38776, USA; 3 Plant Science and Entomology Research Unit, USDA-ARS, Manhattan, KS 66506, USA; 4 Department of Entomology, 202 Plant Industry Building, University of Nebraska, Lincoln, NE 68583, USA; 5 Department of Biochemistry, 141 Chalmers Hall, Kansas State University, Manhattan, KS 66506, USA

## Abstract

**Background:**

Lepidoptera represents more than 160,000 insect species which include some of the most devastating pests of crops, forests, and stored products. However, the genomic information on lepidopteran insects is very limited. Only a few studies have focused on developing expressed sequence tag (EST) libraries from the guts of lepidopteran larvae. Knowledge of the genes that are expressed in the insect gut are crucial for understanding basic physiology of food digestion, their interactions with *Bacillus thuringiensis *(Bt) toxins, and for discovering new targets for novel toxins for use in pest management. This study analyzed the ESTs generated from the larval gut of the European corn borer (ECB, *Ostrinia nubilalis*), one of the most destructive pests of corn in North America and the western world. Our goals were to establish an ECB larval gut-specific EST database as a genomic resource for future research and to explore candidate genes potentially involved in insect-Bt interactions and Bt resistance in ECB.

**Results:**

We constructed two cDNA libraries from the guts of the fifth-instar larvae of ECB and sequenced a total of 15,000 ESTs from these libraries. A total of 12,519 ESTs (83.4%) appeared to be high quality with an average length of 656 bp. These ESTs represented 2,895 unique sequences, including 1,738 singletons and 1,157 contigs. Among the unique sequences, 62.7% encoded putative proteins that shared significant sequence similarities (E-value ≤ 10^-3^)with the sequences available in GenBank. Our EST analysis revealed 52 candidate genes that potentially have roles in Bt toxicity and resistance. These genes encode 18 trypsin-like proteases, 18 chymotrypsin-like proteases, 13 aminopeptidases, 2 alkaline phosphatases and 1 cadherin-like protein. Comparisons of expression profiles of 41 selected candidate genes between Cry1Ab-susceptible and resistant strains of ECB by RT-PCR showed apparently decreased expressions in 2 trypsin-like and 2 chymotrypsin-like protease genes, and 1 aminopeptidase genes in the resistant strain as compared with the susceptible strain. In contrast, the expression of 3 trypsin- like and 3 chymotrypsin-like protease genes, 2 aminopeptidase genes, and 2 alkaline phosphatase genes were increased in the resistant strain. Such differential expressions of the candidate genes may suggest their involvement in Cry1Ab resistance. Indeed, certain trypsin-like and chymotrypsin-like proteases have previously been found to activate or degrade Bt protoxins and toxins, whereas several aminopeptidases, cadherin-like proteins and alkaline phosphatases have been demonstrated to serve as Bt receptor proteins in other insect species.

**Conclusion:**

We developed a relatively large EST database consisting of 12,519 high-quality sequences from a total of 15,000 cDNAs from the larval gut of ECB. To our knowledge, this database represents the largest gut-specific EST database from a lepidopteran pest. Our work provides a foundation for future research to develop an ECB gut-specific DNA microarray which can be used to analyze the global changes of gene expression in response to Bt protoxins/toxins and the genetic difference(s) between Bt- resistant and susceptible strains. Furthermore, we identified 52 candidate genes that may potentially be involved in Bt toxicity and resistance. Differential expressions of 15 out of the 41 selected candidate genes examined by RT-PCR, including 5 genes with apparently decreased expression and 10 with increased expression in Cry1Ab-resistant strain, may help us conclusively identify the candidate genes involved in Bt resistance and provide us with new insights into the mechanism of Cry1Ab resistance in ECB.

## Background

The genomic information on insects has increased tremendously during last several years. Whole genomes have been sequenced for several insect species, including the fruit fly (*Drosophila melanogaster*) [1], African malaria mosquito (*Anopheles gambiae*) [2], yellow fever mosquito (*Aedes aegypti*) [3], honey bee (*Apis mellifera*) [4], silkworm (*Bombyx m*ori) [5,6], red flour beetle (*Tribolium castaneum*) [7], and 11 other *Drosophila *species [8,9]. Genome sequencing of other insect species, including pea aphid (*Acyrthosiphon pisum*), northern house mosquito (*Culex pipiens*), three species of parasitoid wasp (*Nasonia *sp.), Hessian fly (*Mayetiola destructor*), blood sucking bug (*Rhodnius prolixus*), and body louse (*Pediculus humanus*), are currently in progress [10-12]. The red flour beetle is the only agricultural insect pest whose whole genome sequence has become available to date.

Lepidoptera, the second most biodiverse group of insect species after Coleoptera, represents more than 160,000 species including many of the most devastating pests of crops, forests and stored products [13]. The silkworm was the first lepidopteran insect to have its complete genome sequenced [6]. However, genomic information for other lepidopterans, particularly agricultural pest species is limited but urgently needed due to their economic importance and biodiversity. Sequencing of the expressed sequence tags (ESTs) has been recognized as an economical approach to identify a large number of expressed genes that can be used in gene expression and other genomic studies [14-16]. Indeed, ESTs have been generated from several lepidopteran insects including the silkworm [17], spruce budworm (*Choristoneura fumiferana*) [18], cotton bollworm (*Helicoverpa armigera*) [19], diamondback moth (*Plutella xylostella*) [20], tobacco hawkmoth (*Manduca sexta*) [21,22], and fall armyworm (*Spodoptera frugiperda*) [10,23].

It has been long recognized that the insect gut is an important target for developing new strategies for insect pest management. Until now, however, only a few studies have focused on the development of gut-specific EST libraries of lepidopterans as a tool to identify candidate genes involved in the toxicity of insecticides and the development of insecticide resistance. Gut-specific EST libraries were reported for light brown apple moth (*Epiphyas postvittana*) (6,416 ESTs) [24], bertha armyworm (*Mamestra configurata*) (30 serine protease-related sequences) [25], and European corn borer (ECB, *Ostrinia nubilalis*) (1,745 ESTs) [26].

ECB is one of the most destructive pests of corn and can cause as much as $1 billion of economic loss annually in the United States alone [27,28]. ECB also represents a complex of stalk borers, such as the southwestern corn borer (*Diatraea grandiosella*) and the sugarcane borer (*Diatraea saccharalis*). These stalk borers share similar ecosystem and create similar damage to corn plants. Although ECB has been successfully managed using transgenic Bt corn hybrids (plants that express insecticidal toxins of *Bacillus thuringiensis *or Bt), there are increasing concerns about the potential development of Bt resistance in ECB because of the widespread use of Bt corn [28,29]. Indeed, several ECB colonies have developed resistance to Bt toxins under laboratory selection conditions [30,31].

The main target for Bt toxins is the insect midgut, where Bt protoxins are activated by gut proteases to produce activated Bt toxins. The activated toxins then bind to specific receptor(s) to confer toxicity [32]. This means that insect resistance to Bt toxins could be conferred by protease-mediated and receptor-mediated mechanisms [33-37]. Because Bt toxins and insect gut interactions are determined by many gene products in the insect gut, including many proteins/enzymes involved in Bt protoxin activation, toxin binding to receptors and toxin degradation, any change in these systems has the potential to affect a particular Bt's specificity and efficacy, and could lead to Bt resistance in insects.

Our goals are to develop a gut-specific EST database from ECB larvae and explore candidate genes that are potentially involved in insect-Bt interactions and Bt resistance. In this paper, we report the analysis and annotations of 15,000 ESTs derived from the gut of ECB larvae. We discuss the putative identities of the ESTs, their potential biological and molecular functions, and present comparative analyses of our ESTs with sequences from other insects. This work provides the opportunity for developing an ECB gut-specific microarray that can be used to study insect-Bt interactions and genetic basis of Bt resistance in ECB. Furthermore, we revealed 52 candidate genes that could be involved in Bt toxicity and resistance. Among the 41 selected candidate genes examined by RT-PCR, we found 5 genes with apparently decreased expressions and 10 with increased expressions in Cry1Ab-resistant strain of ECB as compared with the susceptible strain of ECB. Differential expressions of these genes in a Cry1Ab-resistant strain may suggest possible involvement of these genes in Cry1Ab resistance, and therefore provides us with new insights into the mechanism of Cry1Ab resistance in ECB. This study may serve as a model for studying Bt resistance mechanisms and for developing bio-pesticides for all closely related corn stalk borers.

## Results and discussion

### Development and analysis of the ECB gut ESTs

We first used pPCR-XL-TOPO plasmid vector to prepare a cDNA library using total RNA purified from the whole guts of fifth-instar larvae of ECB. After we sequenced a total of 1,152 cDNA clones, we found that the cDNA inserts in the vector were not sufficient long (average length: 441 bp). Therefore, we used lambda Uni-ZAP RX vector to prepare a second cDNA library using mRNA purified from the guts of fifth-instar larvae of ECB. This library provided us with much longer cDNA inserts (average length: 674 bp). Because of this significantly improved quality of the ESTs generated from the lambda library, we used the lambda library for our further sequencing of ESTs. Among the 15,000 random cDNA clones sequenced, only <8% were from the plasmid library whereas >92% were from the lambda library (Table 1).

**Table 1:** Summary of the analysis of 15,000 ESTs from the guts of the European corn borer larvae

Library	Sequence direction	Number of clones sequenced	Chromatographs checked (EST number)		Sequence quality checked (EST number)		Average length (bp)	Number of contigs ^*b*^	Number of singletons
							
			Good quality	Poor quality ^*a*^	Good quality	Poor quality			
Plasmid	3'-end	1,152	764	388	722	42	441		
Uni-ZAP RX	5'-end	13,848	12,302	1,546	11,797	505	674		

Total	--	15,000	13,066	1,934	12,519	547	656	1,157	1,738

^*a *^The poor quality sequences were discarded and were not included in the analysis.

^*b *^The numbers of contigs and singletons were based on the analysis of all the ESTs sequenced from the two libraries.

**Figure 1:**
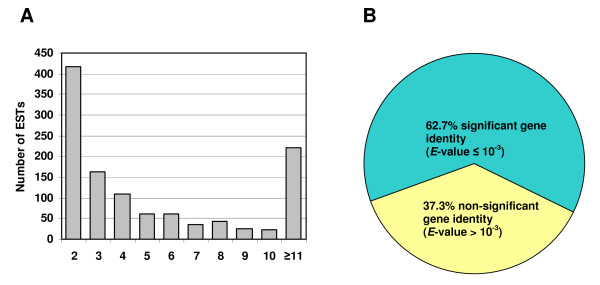
**(A) Distribution of ECB gut-specific ESTs in each contig**. (B) Distribution of the 2,895 ECB gut-specific contigs and singletons with or without match in NCBI database using BLASTx. Sequences were defined as identical or similar cDNA sequences when they had *E*-values ≤ 10^-3^.

Our analysis of the 15,000 sequences resulted in 13,066 readable sequences (i.e., 87.1% success rate). These sequences were first trimmed for removal of vector sequences and then were subjected to filtration to exclude the sequences of <100 bp. Further analysis, using RepeatMasker and Organelle Masker programs [38], removed an additional 547 sequences. Thus, the total number of high quality sequences obtained was 12,519 (83.4%) with an average length of 656 bp (Table 1). These high quality sequences have been deposited in the EST database (dbEST) with GenBank accession numbers from GH987145 to GH999663 at the National Center for Biotechnology Information (NCBI). Redundancy and assembly analyses of the high quality sequences using Sequencher software (Gene Codes Corp., Ann Arbor, MI, USA) resulted in 2,895 unique ESTs, including 1,157 contiguous sequences (contigs) that consist of 2 or more sequences, and 1,738 singletons that represent single sequences. The majority of the contigs were assembled from 10 or fewer ESTs (Figure 1A). On average, however, each contig was assembled from 10.1 sequences due to a few highly redundant ESTs. Putative identities of the unique sequences were determined by searching the non-redundant database in GenBank using BLASTx. Among the 2,895 unique sequences, 1,816 (62.7%) showed significant matches at *E*-values of ≤ 10^-3^, whereas the remaining 1,077 (37.3%) did not exhibit meaningful matches (Figure 1B).

### Transcript abundance

The abundance of transcripts for a particular gene of an organism can be estimated from the corresponding EST abundance in a cDNA library [39]. The most abundant ESTs in our cDNA libraries were those encoding trypsin-like proteases and chymotrypsin-like proteases (Table 2). As this cDNA library was constructed from the gut of ECB, the high number of transcripts from the digestive enzymes was expected. The most abundant contig was #0038 which consisted of 525 ESTs, and it included 4% of the total sequences. This contig shared maximum sequence similarity with the trypsin-like protease, T25 precursor, characterized previously in ECB [40]. Other abundant transcripts were contigs #0026 and #0062. Contig #0026 included 197 ESTs and encoded a putative chymotrypsin-like protease. Contig #0062 included 137 ESTs and encoded a putative trypsin-like serine protease. The highly expressed genes in ECB larval gut may have important implications for their growth and development. However, care must be taken in making general conclusions about the redundancy of EST's because some artifacts could also be involved [41].

**Table 2:** List of 20 largest contigs assembled from 15,000 ESTs from the guts of European corn borer larvae

Contig Identification	Putative identities	Number of ESTs	Length (bp)	% Total	*E*-value
Contig [0038]	Trypsin-like protease T25 precursor	525	942	4.1	3e-148
Contig [0026]	Chymotrypsin-like serine protease	197	1,321	1.5	1e-149
Contig [0062]	Trypsin-like serine protease	132	1,076	1.0	1e-131
Contig [0074]	Unknown	131	824	1.04	--
Contig [0059]	Trypsin-like serine protease	129	1,497	1.0	1e-117
Contig [0076]	Trypsin-like serine protease	129	1,133	1.0	1e-148
Contig [0077]	Unknown	97	652	0.77	--
Contig [0060]	Unknown	94	1,218	0.75	--
Contig [0125]	Ribosomal protein s13	87	888	0.69	1e-79
Contig [0092]	Trypsin-like serine protease	80	1,238	0.63	1e-149
Contig [0102]	Unknown	80	800	0.63	--
Contig [0040]	Thymosin isoform 1	78	1,447	0.62	1e-80
Contig [0243]	Trypsin-like serine protease	78	701	0.62	1e-120
Contig [0034]	Unkown	76	567	0.60	1e-60
Contig [0124]	Pancreatic triacylglycerol lipase	75	1,263	0.59	1e-99
Contig [0426]	Chymotrypsin-like serine protease	73	1,439	0.58	1e-129
Contig [0146]	Unknown	71	839	0.56	--
Contig [0997]	Unknown	71	574	0.56	--
Contig [0013]	Unknown	68	1,244	0.54	--
Contig [0175]	Phosphate mannosyltransferase	65	1,121	0.51	1e-20

### Identification of the ORF and putative secretary proteins

**Figure 2:**
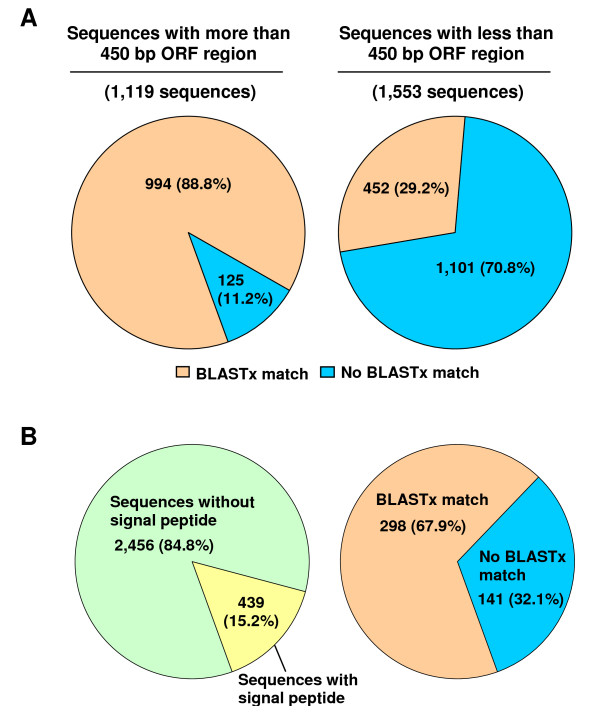
**Open reading frame (ORF), secretory protein, and BLASTx results**. (A) The proportion of the unique ESTs from ECB gut cDNA library with or without 450 bp of ORF region along with their matches in BLASTx using NCBI database. (B) Proportion of the unique ESTs with or without signal peptide along with their match in BLASTx using NCBI database.

The 2,895 contigs and singletons were subjected to the ORF predictor software to identify the open reading frame (ORF) of the sequences. This was done to identify the novel gene candidates, which have clear coding capacity. Among 2,895 unique ESTs, 1,119 (38.7%) had ORFs of at least 450 bp. Among 1,119 ORFs, 994 putative protein sequences (88.8%) shared sequence similarity (E-value ≤ 10^-3) ^with known proteins in the non-redundant (NR) protein database in GenBank, whereas 125 (11.2%) did not share significant similarity with any known protein in the same database (Figure 2A). Thus, at least 11.2% of the protein-coding genes in the gut of ECB are potentially new genes. The remaining 1,553 contigs and singletons (53.6%) had an ORF of <450 bp and 223 (7.7%) did not have an ORF. Among the ESTs with ORFs of <450 bp, 452 (29.2%) had matches in the NR protein database, whereas 1,011 (70.8%) did not have matches. Many sequences did not have ORF of ≥ 450 bp because the sequences were too short (approximately 650 sequences were less than 450 bp). The lack of the ORFs in other sequences can be due to frame shift errors, 5' truncation of cDNA clones and the ESTs that were not derived from mRNA [42].

To identify the secretory proteins, putative protein sequences were examined to identify potential secretion signal peptide using SignalP software [43]. A total of 439 (15.2%) putative proteins were predicted to contain signal peptides (Figure 2B). Among the putative secretory proteins, 298 sequences (67.9%) had matches with known proteins in the NR protein database, whereas 141 putative secretory proteins (32.1%) were unique, sharing no significant sequence similarity with any known protein. This information is valuable since secretory proteins are important components of biological processes in the gut [44,45].

### Comparative analyses of ECB gut ESTs

**Figure 3:**
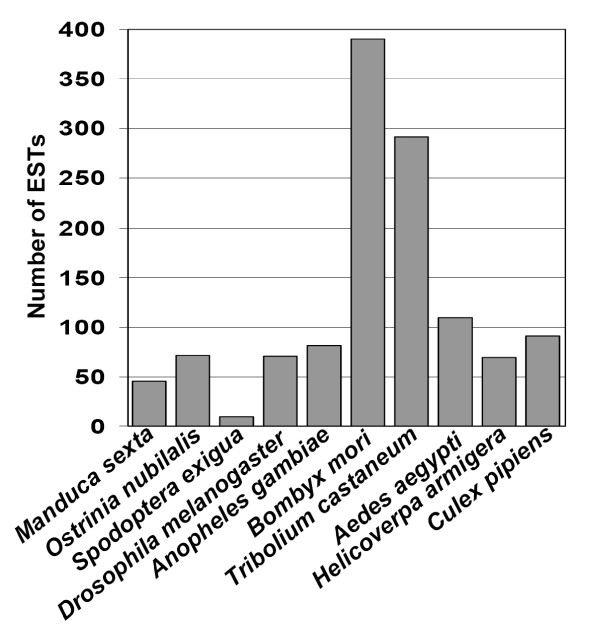
**Similarity of ECB gut-specific ESTs with other insects**. The first hit sequence (highest score) was used to determine the most similar organism.

The development of EST databases has been recognized as a rapid method of sampling an organism's transcriptome and is complementary to a whole genome-sequencing project [46]. Indeed, a large number of ESTs have been generated from other model organisms. The 2,895 contigs and singletons obtained from the larval gut of ECB were compared with the sequences from other organisms. The first hits (highest score) of the sequences in the NR database were taken into account to determine the most similar organism. The largest number of first hit sequences (390; 13.5%) came up with *B. mori *(Figure 3). This can be explained by the fact that the genome of *B. mori *has been sequenced and partially annotated, and that both ECB and *B. mori *are lepidopterans. The second largest number of first hit sequences (290; 10.0%) was with *T. castaneum*, followed by *Ae. aegypti *(109; 3.8%)*, Culex pipiens *(91; 3.1%)*, and A. gambiae *(81; 3.8%). Only 2.5% of the sequences (72) were found to be most similar to predicted protein sequences from *O. nubilalis*. This is simply due to the very small number of sequences currently available in NCBI database from ECB.

**Figure 4:**
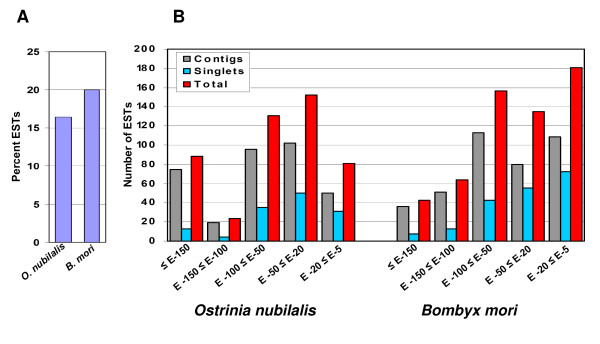
**(A) Percentage of the 2,895 ECB gut-specific unique ESTs which shared matches with *O. nubilalis *and *B. mori *sequences with *E*-value ranging from *E*-150 to *E*-5 using BLASTN**. (B) Comparative analyses of the 2,895 ECB gut-specific unique ESTs to *B. mori *sequences and other *O. nubilalis *sequences available in NCBI database using BLASTN.

In order to compare our ECB gut ESTs with the 1,745 ECB ESTs that are already available in NCBI database, we performed BLASTN searches. Among our 2,895 contigs and singletons, 1,279 (44.2%) had significant matches at a cutoff *E*-value of ≤ 10^-3 ^whereas 1,616 (55.8%) did not show any significant matches in NCBI database using BLASTN search. We compared our ECB ESTs with the ECB ESTs available in NCBI dbEST database. We found 475 sequences (16.4%) that had significant matches with *E*-values less than *E*-150 (Figure 4A). Within this category, 88 ESTs (3.0%) had matches with *E*-values less than *E*-150, 23 (0.8%) had *E*-values between *E*-150 and *E*-100, 131 (4.5%) had *E*-values of *E*-100 and *E*-50, 152 (5.2%) had *E*-values of *E*-50 and *E*-20, and 81 (2.7%) had *E*-values between *E*-20 and *E*-5 (Figure 4B). A total of 2,420 ESTs (83.6%) had no hits with currently available midgut ESTs in NCBI database.

Because *B. mori *genome has not been fully annotated, we have also compared our ESTs with all available *B. mori *ESTs using BLASTN. Among the 2,895 contigs and singletons, 579 (20.0%) had hits with *B. mori *sequences at *E*-value < 10^-3 ^(Figure 4A). The remaining 2,316 ESTs (80.0%) did not show a significant match with the *B. mori *sequences. Among the 579 unique ESTs which had hits in the database, 43 (7.4%) had matches with *E*-value less than *E*-150, 64 (11.1%) had *E*-values between *E*-150 and *E*-100, 156 (26.9%) had *E*-values between *E*-100 and *E*-50, 135 (23.3%) had *E*-values between *E*-50 and *E*-20, and 181 sequences (31.3%) had *E*-values between *E*-20 and *E*-5 (Figure 4B).

### Gene ontology

**Figure 5:**
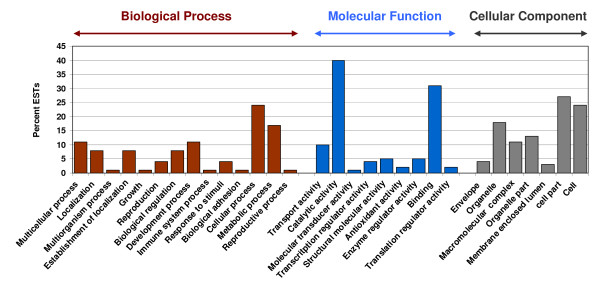
**Distribution of the ECB gut-specific unique ESTs annotated at GO level 2**. The Y-axis shows the percentage of the sequences. The x-axis shows 3 areas of annotation and with each area the sequences were further divided into subgroups at GO level 2.

Blast2GO software was used to obtain the gene ontology (GO) terms for the unique sequences by comparing them through the Gene Ontology Consortium [47]. Among the 2,895 contigs and singletons, 1,815 showed blast hits at *E*-value ≤ 10^-3 ^and 1,119 ESTs of the 1,815 were mapped. A total of 120 mapped ESTs showed both the GO terms and Enzyme Commission (EC) numbers. Figure 5 shows the EST functional categories, where the ECB unique ESTs were assigned to putative biological processes, molecular functions, and cellular components. Within the biological process category, 24.0% belong to cellular processes, followed by 17.0% metabolic processes, 11.0% developmental processes, 11.0% multi-cellular processes, and 8.0% each for biological regulation and localization. In the molecular function category, the maximum GO terms (40.0%) are included in catalytic activity, followed by binding (31.0%), transporter activity (10.0%), and 5.0% each for enzyme regulation activity and structural molecular activity (9.0%). In cellular components category, cell part, cell, and organelle had 27.0%, 24.0%, and 18.0% of the GO terms, respectively. They were followed by organelle part (13.0%), macromolecular complex (11.0%), envelope (4.0%), and membrane-enclosed lumen (3.0%).

### Identification of ESTs potentially relevant to the Bt toxicity and resistance

The mode of Bt action in insects includes the ingestion of Bt protoxins, solubilization of Bt protoxins in insect gut, proteolytic activation of protoxins, binding of toxins to Bt receptors, membrane integration, pore formation, cell lysis, and insect death [48]. According to this mode of action, a target insect could potentially develop resistance to Bt protoxins or toxins via one or more changes in the Bt-receptor interaction pathway. Indeed, the two most commonly identified Bt resistance mechanisms are protease-mediated and receptor-mediated resistance [49]. Our analysis of ESTs derived from the larval gut of ECB revealed a number of genes that are potentially involved in Bt toxicity and resistance (Table 3). Specifically, we identified 18 ESTs putatively encoding trypsin-like proteases and 18 ESTs putatively encoding chymotrypsin-like proteases with *E*-value ranges from 2e-26 to 3e-137 and *E*-value 3e-27 to 3e-149, respectively. Changes in the proteolytic activity of digestive enzymes can alter the toxicity of Bt protoxins or toxins through effects on crystal solubilization and/or activation of protoxins, as well as degradation of activated toxin [33,50-56]. A previous study from our lab has shown that Bt resistance in a Dipel-resistant strain of ECB was primarily associated with reduced trypsin-like protease activity [35,40]. These trypsin-like proteases were also revealed in our EST analysis. Thus, our analysis of the ESTs generated from the guts of ECB larvae revealed many more candidate genes that deserve further analysis for their roles in Bt toxicity and resistance in ECB.

**Table 3:** List of genes potentially involved in Bt toxicity and resistance as identified by EST analysis from the guts of the European corn borer larvae

European corn borer							Silkworm^*b*^	
	
	EST ID	Matches	Organism^*a*^	% Identities	*E*-value	Matches	% Identities	*E*-value
**Trypsin-like serine proteases**								
1	Contig [0038]	AAR98918.1	*Ostrinia nubilalis*	254/256 (99%)	3e-148	AAB26023.1	144/233 (61%)	2e-78
2	Contig [0157]	ACB54937.1	*Helicoverpa armigera*	46/97 (47%)	8e-18	No match	--	--
3	Contig [0111]	ABF47507.1	*Ostrinia furnacalis*	248/257 (96%)	5e-135	AAB26023.1	136/231 (58%)	2e-64
4	Contig [0291]	AAX62039.1	*Ostrinia nubilalis*	257/258 (99%)	5e-137	AAB26023.1	100/240 (41%)	1e-43
5	Contig [0486]	ABU98624.1	*Helicoverpa armigera*	148/241 (61%)	1e-72	AAB26023.1	109/235 (46%)	4e-48
6	Contig [0754]	AAX62034.1	*Ostrinia nubilalis*	154/266 (57%)	7e-69	AAB26023.1	128/244 (52%)	3e-56
7	Contig [0622]	ABU98624.1	*Helicoverpa armigera*	145/249 (58%)	4e-82	AAB26023.1	101/238 (42%)	3e-50
8	Contig [0907]	ABU98619.1	*Helicoverpa armigera*	89/206 (43%)	6e-47	No match	--	--
9	Contig [1007]	AAR98918.1	*Ostrinia nubilalis*	218/252 (86%)	1e-120	AAB26023.1	119/229 (51%)	1e-57
10	Contig [1400]	ABU98619.1	*Helicoverpa armigera*	56/199 (28%)	2e-21	No match	--	--
11	Contig [1615]	ABF47507.1	*Ostrinia furnacalis*	101/189 (53%)	1e-64	AAB26023.1	95/183 (51%)	5e-50
12	Contig [3395]	AAX62032.1	*Ostrinia nubilalis*	129/209 (61%)	3e-72	AAB26023.1	117/208 (56%)	7e-59
13	Contig [4300]	AAX62035.1	*Ostrinia nubilalis*	79/84 (94%)	8e-43	AAB26023.1	43/72 (59%)	5e-16
14	Contig [4291]	AAX62032.1	*Ostrinia nubilalis*	181/236 (76%)	2e-105	AAB26023.1	138/233 (59%)	7e-75
15	ECB-30_C08	AAX62036.1	*Ostrinia nubilalis*	36/45 (80%)	1e-13	No match	--	--
16	ECB-17_C09	ABC87051.1	*Ostrinia furnacalis*	65/98 (66%)	5e-30	AAB26023.1	48/97 (49%)	4e-19
17	ECB-C-18_B11	AAR98920.2	*Ostrinia nubilalis*	198/204 (97%)	5e-114	No match	--	--
18	ECB-V-26_H09	ABC87051.1	*Ostrinia furnacalis*	35/50 (70%)	6e-12	NP_001040350	24/48 (50%)	3e-04
**Chymorypsin-like serine proteases**								
1	Contig [0026]	AAX62029.1	*Ostrinia nubilalis*	258/261 (98%)	5e-149	NP_001036903.1	163/259 (62%)	1e-86
2	Contig [0058]	AAX62029.1	*Ostrinia nubilalis*	228/261 (87%)	3e-120	No match	--	--
3	Contig [0120]	AAF71515.1	*Agrotis ipsilon*	174/287 (60%)	2e-84	NP_001040430.1	109/244 (44%)	2e-47
4	Contig [0141]	AAX62028.1	*Ostrinia nubilalis*	197/262 (75%)	1e-101	NP_001040430.1	130/261 (49%)	2e-52
5	Contig [0187]	AAX62026.1	*Ostrinia nubilalis*	193/202 (95%)	4e-97	No match	--	--
6	Contig [0299]	AAX62029.1	*Ostrinia nubilalis*	228/261 (87%)	6e-120	NP_001036903.1	166/259 (64%)	7e-82
7	Contig [0379]	AAX62030.1	*Ostrinia nubilalis*	111/242 (45%)	1e-55	NP_001036903.1	109/236 (46%)	8e-55
8	Contig [0426]	AAX62026.1	*Ostrinia nubilalis*	282/289 (97%)	1e-129	No match	--	--
9	Contig [0560]	NP_001040430.1	*Ostrinia nubilalis*	128/232 (55%)	1e-63	NP_001040430.1	171/272 (62%)	4e-93
10	Contig [0806]	AAX62029.1	*Ostrinia nubilalis*	202/208 (97%)	6e-137	No match	--	--
11	Contig [1061]	CAL92020.1	*Manduca sexta*	169/281 (60%)	1e-87	No match	--	--
12	Contig [1478]	NP_001040430.1	*Bombyx mori*	152/260 (58%)	5e-84	NP_001040430.1	152/260 (58%)	4e-84
13	Contig [2079]	AAL93243.1	*Aedes aegypti*	85/242 (35%)	7e-40	No match	--	--
14	Contig [2569]	AAF71518.1	*Helicoverpa zea*	119/240 (49%)	7e-49	NP_001040430.1	87/212 (41%)	1e-30
15	Contig [4479]	AAC36150.1	*Plodia interpunctella*	140/263 (53%)	3e-77	NP_001036826.1	117/270 (43%)	3e-51
16	Contig [4699]	AAX62029.1	*Ostrinia nubilalis*	195/261 (74%)	1e-102	NP_001036826.1	144/251 (57%)	9e-68
17	ECB-23_F02	CAM84318.1	*Manduca sexta*	88/209 (42%)	3e-36	No match	--	--
18	ECB-V-25_E02	AAX62031.1	*Ostrinia nubilalis*	32/32 (100%)	2e-11	NP_001040430.1	99/203 (48%)	6e-42
**Aminopeptidases**								
1	Contig [0722]	AAP37951.1	*Helicoverpa armigera*	72/193 (37%)	9e-29	BAA33715.1	60/160(37%)	2e-21
2	Contig [1364]	ABL01481.1	*Ostrinia nubilalis*	413/421 (98%)	0.0	NP_001037013.1	273/422 (64%)	2e-154
3	Contig [1716]	XP_560264.3	*Anopheles gambiae*	132/354 (37%)	3e-59	No match	--	--
4	Contig [1907]	ACB87202.1	*Ostrinia furnacalis*	370/374 (98%)	0.0	BAA33715.1	240/368 (65%)	8e-135
5	Contig [4362]	AAQ57405.1	*Helicoverpa armigera*	102/263 (38%)	2e-48	NP_001037013.1	89/266 (33%)	1e-42
6	Contig [4298]	ACB47287.1	*Ostrinia furnacalis*	291/297 (97%)	3e-167	NP_001036834.1	213/297 (71%)	6e-118
7	Contig [4992]	AAP37951.1	*Helicoverpa armigera*	168/246 (68%)	7e-99	BAA33715.1	85/238 (35%)	6e-38
8	Contig [4529]	ABV01346.1	*Ostrinia furnacalis*	342/356 (96%)	0.0	NP_001104835.1	226/350 (64%)	4e-125
9	ECB-G02	AAK85539.1	*Helicoverpa armigera*	196/262 (74%)	2e-114	No match	--	--
10	ECB-D07	ABQ51393.1	*Ostrinia furnacalis*	171/22 (75%)	1e-99	NP_001104835.1	159/225 (70%)	2e-95
11	ECB-D12	ABV01346.1	*Ostrinia furnacalis*	200/278 (71%)	1e-112	NP_001104835.1	155/275 (56%)	5e-80
12	ECB-C06	ABL01481.1	*Ostrinia nubilalis*	40/40 (100%)	9e-16	NP_001037013.1	23/39 (58%)	7e-07
13	ECB-F04	AAP37951.1	*Helicoverpa armigera*	117/208 (56%)	2e-61	BAA32475.1	67/190 (35%)	2e-24
**Alkaline phosphatases**								
1	Contig [5091]	NP_001037536.2	*Bombyx mori*	101/172 (58%)	3e-53	NP_001037536.2	101/172 (58%)	3e-53
2	Contig [2328]	BAF62124.1	*Bombyx mandarina*	176/260 (67%)	8e-103	NP_001037536.2	177/260 (68%)	2e-102
**Cadherin-like protein**								
1	ECB-B09	ABS59299.1	*Ostrinia furnacalis*	242/244 (99%)	7e-135	BAA99405.1	155/247 (62%)	1e-81

^*a *^Sequence with highest score in BLASTX search

^*b *^Match of ECB ESTs with silkworm sequence using BLASTX

Our EST analysis also revealed 13 ESTs putatively encoding aminopeptidases (*E*-value 1e-64 to 1e-116), 1 encoding a cadherin-like protein (*E*-value 1e-35), and 2 encoding alkaline phosphatases (*E*-value 1e-115 to 1e-131). Aminopeptidase N, cadherin-like proteins, and alkaline phosphatases have been found to serve as Bt toxin binding receptors in other insect species [57-59]. To verify the function of aminopeptidase N as a receptor for Bt Cry1Ac toxin in *Spodoptera litura*, RNAi technology was used to reduce the expression of aminopeptidase N. This resulted in a significant reduction in the susceptibility of the insect to Cry1Ac toxin [60]. Gahan *et al*. [61] showed that in a resistant strain (YHD2) of *Heliothis virescens*, there was a disruption of a cadherin-superfamily gene by a retrotransposon-mediated insertion that resulted in high levels of resistance to the Bt toxin Cry1Ac. Fernandez *et al*. [62] also reported that a GPI (glycosylphosphatidyl-inositol)-anchored ALP (alkaline phosphatase) was an important receptor molecule involved in Cry11Aa interactions with midgut cells and toxicity to *Ae. aegypti *larvae. These studies demonstrate that aminopeptidases, cadherin-like proteins, and alkaline phosphatases can serve as Bt toxin receptors involved in Bt toxicity and resistance. Thus, identification of these candidate Bt receptor genes in this study will allow us to further examine whether receptor-mediated resistance is involved in Bt resistance in ECB.

### Comparison of expression profiles between Cry1Ab-susceptible and resistant strains of ECB

**Figure 6:**
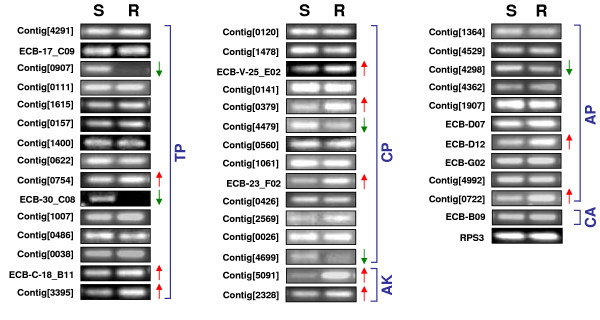
**Expression profiles of 41 candidate genes relevant to Bt toxicity and resistance, which include 15 trypsin-like serine protease (TP), 13 chymotrypsin-like serine protease (CP), 2 alkaline phosphatase (AK), 10 aminopeptidase (AP), and 1 cadherin-like protein (CA) genes in the midgut of one-day-old fifth-instar larvae in Cry1Ab-susceptible (S) and resistant (R) strains of ECB as determined by RT-PCR**. The arrows next to the gel pictures indicate the apparently decreased (↓) or increased (↑) expression of a particular gene in Cry1Ab-resistant strain as compared with the susceptible strain of ECB. The ribosomal S3 (*RPS3*) gene was used as a reference gene. At least two biological replications were used for each primer pair. The sequences of PCR primers used in this study were given in Appendix A.

We performed RT-PCR to compare the expression patterns of the candidate genes relevant to Bt toxicity and resistance between Cry1Ab-susceptible and resistant strains of ECB. Among 41 selected genes from the 52 candidate genes, which included 15 that putatively code for trypsin-like serine proteases, 13 for chymotrypsin-like serine proteases, 10 for aminopeptidases, 2 for alkaline phosphatases, and 1 for cadherin-like protein, we found apparently decreased expressions in 2 trypsin-like and 2 chymotrypsin-like protease genes, and 1 aminopeptidase genes in the resistant strain as compared with the susceptible strain (Figure 6). Among these genes, 2 trypsin-like protease genes (contig [0907] and ECB-30-C08) were virtually absent in the resistant strain. In contrast, we found apparently increased expressions in 3 trypsin-like and 3 chymotrypsin-like protease genes, 2 aminopeptidase genes, and 2 alkaline phosphatase genes in the resistant strain. The most noticeable increases were found in 1 trypsin-like protease (contig [3395]), 3 chymotrypsin-like protease (ECB-V-25_E02, contig [0379], and ECB-23_F02), 1 alkaline phosphatase (contig [5091]), and 1 aminopeptidase (ECB-D12) genes.

Although RT-PCR is not quantitative, reproducible results of such differential expression patterns for these candidate genes in the Cry1Ab-susceptible and resistant strains of ECB may imply their potential roles in conferring or contributing to Cry1Ab resistance as well as genetic differences between the susceptible and resistant strains of ECB. Indeed, certain trypsin-like and chymotrypsin-like proteases have previously been found to activate or degrade Bt protoxins and toxins, whereas several aminopeptidases, cadherin-like proteins and alkaline phosphatases have been demonstrated to serve as Bt receptor proteins in other insect species. Thus, our results may help conclusively identify the candidate genes involved in Cry1Ab resistance and provide us with new insights into the mechanism of Cry1Ab resistance in ECB. Nevertheless, further research will be needed to confirm their involvements and to elucidate their roles in Cry1Ab resistance in ECB.

## Conclusion

Our study resulted in a gut-specific EST database containing 12,519 high-quality ESTs from a total of 15,000 ESTs sequenced in an agriculturally important lepidopteran pest. To our knowledge, this database represents the largest gut-specific EST database from a lepidopteran pest. Our analysis using ORF predictor software showed that approximately 11.2% of the protein coding genes in our database may be specific to ECB as these sequences have an ORF of at least 450 bp but did not have significant matches with known sequences in NCBI database. We have also identified 52 candidate genes that are relevant to Bt toxicity and resistance. These genes encode trypsin-like proteases, chymotrypsin-like proteases, aminopeptidases, cadherin-like protein, and alkaline phosphatases. Furthermore, we showed differential expressions of 15 out of the 41 representative candidate genes that were examined by RT-PCR, including 5 genes with apparently decreased expressions and 10 with increased expressions in Cry1Ab-resistant strain. These results may help us further narrow down the candidate genes possibly involved in Cry1Ab resistance, and provide us with new insights into the mechanism of Bt resistance in general in ECB.

We are in the process of developing a microarray using our unique ESTs together with the ECB gut-specific sequences which are already available in the GenBank. The microarray technology will help us analyze the global change of gene expression in response to Bt protoxins/toxins. It will also allow us to analyze any genetic differences between Bt resistant and -susceptible strains of ECB. Our genomic information on ECB could also serve as a valuable resource for identifying critical/vulnerable genes from the gut of ECB that would make useful physiological targets for new toxins that could be developed for use in pest management.

## Methods

### Insects rearing and dissection

The KS-SC Bt-susceptible ECB colony was used for generating EST libraries. This colony originated from the egg masses collected from the cornfields near St. John, Kansas, in 1995. The colony has been reared since then on artificial diets in the laboratory at Kansas State University according to Huang *et al*. [63]. The resistant ECB strain originated from a field collection of 126 diapausing larvae obtained from non-Bt hybrids in Kandiyohi Co., MN in 2001. The resistant strain was initiated from 14 larvae that survived exposure to a diagnostic Cry1Ab concentration used to identify potential changes in susceptibility to Cry1Ab [64,65]. To minimize inbreeding or founder effects, the resistant insects were backcrossed twice with the susceptible strain which originated from the same collection. Because the resistance was incompletely recessive and involved multiple factors [65], the F_1 _progeny were randomly mated to obtain recombination of resistance factors in the F_2 _progeny to allow selection of resistant genotypes. The insects were then subjected to selection at a Cry1Ab concentration corresponding to two- to three-fold the LC_50 _for the F_1 _progeny (150 ng/cm^2^) [66]. This selection event was designed to eliminate all the susceptible homozygotes and most of the heterozygotes. The resistant survivors from this selection event were then subjected to a second cycle of backcrossing, random mating, and selection. After six generations, the Cry1Ab concentration used in selections was gradually increased to achieve 750 ng/cm^2 ^at generation F_10_, a concentration that kills virtually all F_1 _progeny. At generation F_17_, the resistance to Cry1Ab in the re-selected strain was in excess of 800-fold. The guts were dissected from fifth-instar larvae in DEPC (diethylpyrocarbonate)-treated distilled water and were stored in TRI reagent™ (Molecular Research, Inc., Cincinnati, OH) at -80°C until used.

### cDNA library construction and sequencing

Total RNA was isolated from the whole guts of ECB larvae using TRI reagent™. The plasmid library was constructed using Creator SMART™ cDNA library construction kit from Clontech (Palo Alto, CA) following the manufacturer's protocols with one modification; instead of using the original phage vector, PCR fragments were cloned directly into a pPCR-XL-TOPO plasmid using a TOPO TA cloning kit (Invitrogen, Carlsbad, CA). The λ-library was constructed using ZAP-cDNA synthesis kit and ZAP-cDNA Gigapack III gold cloning kit (Stratagene, La Jolla, CA) according to the manufacturer's protocols. Briefly, double stranded cDNA was synthesized from poly(A) RNA, size-fractionated through a Sepharose CL-2B gel filtration column, and ligated into λ Uni-ZAP XR vector. The ligated DNA was packaged with the Gigapack III gold packaging extract and the library was plated on LB/agar plates. Recombinant plasmid within the lambda Uni-ZAP XR vector was *in vivo *excised using the ExAssist helper phage and recircularized to generate subclones in the pBluescript SK phagemid vector. To sequence the clones, M13R and M13F primers were used for 5' and 3' sequencing, respectively. Plasmid DNA was isolated using Qiagen Bio Robot 3000 and sequenced using an ABI 3700 DNA analyzer.

### EST analyses and annotations

The DNA sequences were preprocessed by using the online software EGassembler [38]. Specifically, sequence cleaning process was employed to trim the vector and adaptor sequences from the ESTs. RepeatMasker process was used to mask the interspersed repeats and low complexity regions of the sequences by using *Drosophila *Repbase repeat library. The sequences were further masked by using vector masking against NCBI's vector library and organelle masking against mitochondrial library. The preprocessed ESTs were then assembled by using Sequencher software (Gene Codes Corp., Ann Arbor, MI). The ORF regions of the assembled ESTs were identified by using the ORF predictor software [67] and secretory proteins were identified by looking for signal peptide sequence using SignalP software [43]. Gene ontology (GO) annotation was derived using Blast2GO software [68].

### Comparative analysis of ESTs

The ECB unique ESTs were comparatively analyzed for their sequence similarities against other organisms. The organism associated with the EST showing the highest BLAST score in GenBank databases was selected. The ECB gut ESTs were also compared with sequences from the silkworm and ECB that are currently available in the database by using BLASTN with a cutoff *E*-value of 10^-3^.

### Expression profiling by RT-PCR

Forty-one out of the 52 candidate genes were selected for comparing their apparent gene expression profiles between the Cry1Ab-susceptible and resistant strains of ECB by using RT-PCR. These genes were selected solely based on their representations among different gene groups from our EST analysis. After total RNA was isolated from four midguts dissected from one-day-old fifth-instar larvae of each strain (Cry1Ab-susceptible and resistant strains) of ECB by using TRI reagentTM (Sigma, St. Louis, MO), it was treated with TURBO™ DNase (Ambion, Austin, TX)to remove any genomic DNA contaminations. Three micrograms of total RNA was used for synthesis of first strand cDNA using SuperScript^® ^III First-Strand Synthesis System (Invitrogen, Carlsbad, CA). cDNA prepared from total RNA was used as a template for RT-PCR. A minimum of two biological replications was used for all the PCR primer pairs. For all trypsin-like (except for ECB-30_C08) and chymotrypsin-like serine protease, alkaline phosphatase, and RPS3 genes, 25 PCR cycles were used whereas for aminopeptidase and cadherin-like protein, 27 PCR cycles were used. For one trypsin-like serine protease gene (ECB-30_C08), however, 33 PCR cycles were used as the expression of this gene using fewer cycles was not visible on agarose gels. Each PCR was performed for above mentioned number of cycles, each consisting of 94°C for 30s, 55°C for 60s, and 72°C for 60s. The sequences of forward and reverse PCR primers, and expected size of PCR product for each of 41 candidate genes are provided in Additional file 1.

## Authors' contributions

CK conducted the major part of this study including experimental design, construction of the cDNA libraries, EST analysis, RT-PCR analysis, and manuscript preparation. YCZ participated in experimental design, EST sequencing and preliminary analysis of EST data. MSC assisted in the development of the project, the establishment of the collaboration in EST sequencing, and manuscript preparation. LLB participated in experimental design, maintenance of the insect culture, and manuscript preparation. RAH participated in the development of the project and experimental design. JY assisted in EST sequencing and analysis. BDS and ALBC contributed materials and participated in data analysis and manuscript preparation. SM participated in experimental design and manuscript preparation. KYZ coordinated the project and participated in experimental design, EST analysis, and manuscript preparation. All authors read and approved the final manuscript.

## Supplementary Material

Additional file 1 **Sequences of PCR primers used to compare the gene expression profiles of trypsin-like and chymotrypsin-like serine proteases, alkaline phosphatases, aminopeptidases, and cadherin-like protein by RT-PCR between Cry1Ab-susceptible and resistant strains of European corn borer (*Ostrinia nubilalis*)**. The information provided represents the sequences of forward and reverse PCR primers, and expected size of PCR product for each of 41 candidate genes. Click here for file
