# Supplementary material for: Expressed sequence tags from larval gut of the European corn borer (Ostrinia nubilalis): Exploring candidate genes potentially involved in Bacillus thuringiensis toxicity and resistance
Source: BMC Genomics. 2009 Jun 29;10:286. doi: 10.1186/1471-2164-10-286 (PMC2717985; doi:10.1186/1471-2164-10-286)
Supplement: Additional file 1 — Sequences of PCR primers used to compare the gene expression profiles of trypsin-like and chymotrypsin-like serine proteases, alkaline phosphatases, aminopeptidases, and cadherin-like protein by RT-PCR between Cry1Ab-susceptible and resistant strains of European corn borer (Ostrinia nubilalis). The information provided represents the sequences of forward and reverse PCR primers, and expected size of PCR product for each of 41 candidate genes. [file 1471-2164-10-286-S1.doc]

**Additional File**

**Sequences of PCR primers used to compare the gene expression profiles of trypsin-like and chymotrypsin-like serine proteases, alkaline phosphatases, aminopeptidases, and cadherin-like protein by RT-PCR between Cry1Ab-susceptible and resistant strains of European corn borer (*Ostrinia nubilalis*)**

| Name | Forward Primer | Reverse Primer | Product size (bp) |
| --- | --- | --- | --- |
| **Trypsin-like serine proteases** | | | |
| Contig[0111]  Contig[0486]  Contig[0754]  Contig[0622]  Contig[0907]  Contig[1007]  Contig[1400]  Contig[1615]  Contig[3395]  Contig[0157]  Contig[4291]  Contig[0038]  ECB-30_C08  ECB-17_C09  ECB-C-18_B11 | ACCTGTCCATCATCCGAACC  ATGGCGTCCTCGTTGGTG  TGGGACTGTCTACACTATTGAAAG  CTGGTGGAGTTATTGCCTACG  GGCTACTCCTGCGGTCAC  ATGCGTACCTTCATCGTTCTAC  ACGGAAGGTGGCACTCTC  ACCAGTTCACCAGGGACAAC  TGCTGGTGACTCAAACTCAATG  GCCAGCATTACACCTTCCG  CTCAACAACCGTGCTATCCTC  CATCACGGAGAACATGCTTTG  GATCACCATTTTGGAATTTTCG  TGTTTCATCGGTACTGTCACTG  CACAAAGTCCTGGAGGAAGATTC | TCAGACGACGATCCTCCTTG  TGGTGCCTCCCACAATGC  GATGTGACGGGTATGATGCC  GTGGTTTGCTGGATGGATGG  CTGGACTGCTGCTGTATTGG  GCCATCTCAGGGTATTGGTTAATG  TCTCTTGCGGAGGGATGTAG  TGATGCTGCCAGGGATGAC  TGATGACTCGGTTCAAATAGCG  TCGCAGTTCTCGTAGTAAGAC  GCAGTGTTAATTACAGTTCCATCG  CGTTGACACCAGGGAAGAAG  GAGATACACGGGCGTTGC  GAGGATCACTCGTCTGTTAAGG  GTTCACGCCTGTCTGTTGC | 157  82  120  133  103  116  154  87  101  128  119  158  192  193  125 |
| **Chymotrypsin-like serine proteases** | | | |
| Contig[0026]  Contig[0120]  Contig[0141]  Contig[0426]  Contig[0560]  Contig[1061]  Contig[1478]  Contig[2569]  Contig[4479]  Contig[0379]  Contig[4699]  ECB-23_F02  ECB-V-25_E02 | GAGGAGGGCACGGACTTC  TGTGATCCAGCCCATCTCTC  GCTGGTTCCCTCTACTGGTC  ACCTGCCTACCAGCGTTTC  TCAGTGGAACCCGTGGAAC  TCCTCGCCTGTGGTGTTC  GCCGCTGGATTTGGAAAGAC  TGCTTCTGGATTCGGAATGAC  TTGCGGGATACGGGAAGAC  CCTACTGAGGATGCGAATAACG  CGTCCCTCTTGTGACAATGAAG  TGGTGGAGCCTCTATCATCAG  ATCACCGCTGCTCATTGC | TTCCTGTGTTCAAGGTGATGAC  CAGAAGTGCGTCCGAATCC  GAGATGGTGTTGGAGAAGGC  CCGAAGCCTGAAGCAATAGC  CAGTGCGATTGGTTGGATGG  GATGGTGGTCACGGTCAAC  GAGGGTGCTCGGGAATACG  GGAGATGACTGGAAGAGTAACG  GGAGATTGACCGAGTGGAGAG  TGGGTTGGCTGGGTTTGG  CCAGATCCTGCTGCCATCG  GATTGCCATTCGTTGGTTGC  ACTCCTCCGCTGAAGATGG | 106  95  79  112  94  156  135  85  75  96  92  129  92 |
| **Aminopeptidases** | | |  |
| Contig[0722]  Contig[1364]  Contig[1907]  Contig[4362]  Contig[4298]  Contig[4292]  Contig[4529]  ECB-G02  ECB-D07  ECB-D12 | GCACCCCATTCATTGTTCGC  TCTGTAGTCTGGTTCACATTATCC  AATTCCAAACCTGGGCGTAC  ATCTGAAAAGCACCAACAGTCTTC  ACCCTAACAGTAAGACAGTTTGAC  AAGTCGTAAAGAGTAAACTGAGAG  CTTCAACAGCCCACTGGAGAG  CGACTGGTTCAGGTATTGGTTC  CGCCGTGACCGTAACTGG  TGTATTGGCGGAGTCTGATTC | GTATCTGGACGAGCCTGGAC  ACTCACCTCCGCTGTATCC  GTTGTTCATGGCACTGTTGAC  CTCTCGCCCTGATCGTCTTATG  TGGCACTACAAGCAAGTAACG  GCCAGATCCAGCATGAAGTG  ACGCAAGACATATTAGGTAACAGC  AGGGTGATGCTTCAGACTACG  GTCGTCGCTAACAGAGAAGAG  CCAGTCGTCATTGAGGAACC | 126  84  89  156  197  112  85  137  195  93 |
| **Alkaline phosphatases** | | |  |
| Contig[5091]  Contig[2328] | ACTCGCTCATCGTGGTCAC  CGGATTATCTGCTGGGTTTATTTG | GTCGTCCTCCGTCGTCAC  AGTGTGGGCTCGGTAACG | 200  79 |
| **Cadherin-like protein** | | |  |
| ECB-B09 | GGTCATCAGCACGAAGAG | CAAGCATAGATACTAAGAACTGG | 176 |
